# Improving Children’s Mental Health Literacy Through the Cocreation of an Intervention and Scale Validation: Protocol for the CHILD-Mental Health Literacy Research Study

**DOI:** 10.2196/51096

**Published:** 2023-10-05

**Authors:** Florence Francis-Oliviero, Céline Loubières, Christine Grové, Alexandra Marinucci, Rebecca Shankland, Réda Salamon, Emmanuelle Perez, Laure Garancher, Cédric Galera, Elsa Gaillard, Massimiliano Orri, Juan Luis González-Caballero, Ilaria Montagni

**Affiliations:** 1 Bordeaux Population Health Research Center Institut national de la santé et de la recherche médicale U1219 University of Bordeaux Bordeaux France; 2 Pôle de santé publique Service d’Information Médicale Centre Hospitalier Universitaire de Bordeaux Bordeaux France; 3 Psycom - Santé mentale Info Paris France; 4 School of Curriculum Teaching & Inclusive Education Monash University Clayton Australia; 5 Fulbright Association Science, Technology, Engineering and Mathematics College Health and Biomedical Sciences, Royal Melbourne Institute of Technology Canberra Australia; 6 School of Educational Psychology & Counselling Monash University Clayton Australia; 7 Laboratory Développement, Individu, Processus, Handicap, Éducation Department of Psychology, Education and Vulnerabilities Université Lumière Lyon 2 Lyon France; 8 The Ink Link Nongovernmental organization Lyon France; 9 The Ink Link Nongovernmental organization Paris France; 10 Charles Perrens Hospital Bordeaux France; 11 Institute of Global Health University of Geneva Geneva Switzerland; 12 McGill Group for Suicide Studies Douglas Mental Health University Institute, Department of Psychiatry McGill University Montréal, QC Canada; 13 Department of Epidemiology, Biostatistics, and Occupational Health School of Population and Global Health McGill University Montréal, QC Canada; 14 Department of Statistics and Operational Research University of Cádiz Cádiz Spain

**Keywords:** child, mental health, literacy, intervention, scale

## Abstract

**Background:**

Children’s mental health is a public health priority, with 1 in 5 European children younger than 12 years having a behavioral, developmental, or psychological disorder. Mental health literacy (MHL) is a modifiable determinant of mental health, promoting psychological well-being and reducing mental health problems. Despite its significance, no interventions or scales currently exist for increasing and measuring MHL in this population.

**Objective:**

This study has dual objectives: (1) cocreating and evaluating an intervention on children’s MHL, and (2) developing and validating a scale that measures children’s MHL.

**Methods:**

Our study focuses on children aged 9-11 years attending primary school classes in various settings, including urban and rural areas, and priority education zones within a French department. Using a participatory research approach, we will conduct workshops involving children, parents, teachers, and 1 artist to cocreate an intervention comprising multiple tools (eg, a pedagogical kit and videos). This intervention will undergo initial evaluation in 4 classes through observations, interviews, and satisfaction questionnaires to assess its viability. Concurrently, the artist will collaborate with children to create the initial version of the CHILD-MHL scale, which will then be administered to 300 children. Psychometric analyses will validate the scale. Subsequently, we will conduct a cluster randomized controlled trial involving a minimum of 20 classes, using the CHILD-MHL scale scores as the primary end point to evaluate the intervention’s efficacy. Additional interviews will complement this mixed methods evaluation. Both the intervention and the scale are grounded in the Child-Focused MHL model.

**Results:**

The first tool of the intervention is the pedagogical kit Le Jardin du Dedans, supported by the public organization Psycom Santé Mentale Info and endorsed by UNICEF (United Nations Children’s Fund) France. The second tool is a handbook by the Pan American Health Organization and the World Health Organization, which is addressed to teachers to sensitize them to children’s mental health problems. The third is a 5-page supplementary leaflet produced by the nongovernmental organization The Ink Link, which teaches children the notion of MHL. Finally, we produced 56 items of the MHL Scale and listed existing education policies for children’s mental health.

**Conclusions:**

After its robust evaluation, the intervention could be extended to several schools in France. The scale will be the first in the world to measure children’s MHL. It will be used not only to evaluate interventions but also to provide data for decision makers to include MHL in all educational policies. Both the intervention and the scale could be translated into other languages.

**International Registered Report Identifier (IRRID):**

PRR1-10.2196/51096

## Introduction

### Children’s Mental Health

Children’s mental health is a public health priority [1]. Promoting their mental health means strengthening their capacity to learn and build meaningful relationships. Good mental health during childhood strongly contributes to positive outcomes in adulthood [2]. It is during this period of life that we learn to reinforce protective factors, as well as internal and external resources. A good home environment, convenient conditions at school, a healthy lifestyle, child protection policies, and adequate mental health services promote children’s mental health [3]. Without mental health, there can be no physical health [4]. Nevertheless, children’s mental health has been largely ignored, including by public policies [5].

Only recently, in the aftermath of the COVID-19 pandemic, children’s mental health has been put under the spotlight. Repeated lockdowns, movement restrictions, and school closures have had an undeniable negative impact on children’s mental health status. Statistics report that levels of anxiety and depression in preadolescents increased by 57.4% and 39.3%, respectively [6]. However, the pandemic has been the tip of the iceberg when it comes to poor mental health outcomes in children who represent an undoubtedly vulnerable population.

Previous studies report alarming statistics: in Europe, 1 child out of 5 is affected by a mental health problem before the age of 12 years [7]. While the prevalence of mental disorders is unstable across national and international studies, all statistics confirm that children are especially suffering from, in order of frequency, anxiety, attention-deficit/hyperactivity disorder, depression, learning disability, and autism [8]. Their onset can be detected between ages 3 and 11 years [9]. These problems have irreversible consequences on adulthood if they are not treated early, even leading to premature death [10]. In fact, 50% of adult mental illnesses have their origins in childhood [11].

Paradoxically, previous studies report that mental health problems in children are often underdiagnosed and untreated. Their signs and symptoms can be difficult to detect, thus delaying appropriate access to care.

When talking about children’s mental health, it is important not to reduce it to problems or illness, but to help children adopt and maintain healthy lifestyles and provide supportive living conditions and environments. Accordingly, to increase children’s psychological well-being, mental health promotion strategies have been encouraged worldwide [12,13]. The objective was to improve resilience and develop psychosocial skills so that children could learn to overcome adversity and enhance positive mental health through the encouragement and increase of protective factors and healthy behaviors.

### The Population Under Study

Among different childhood stages, mid-late childhood or preadolescence (9-11 years old) deserves attention as it is a vulnerable transition period during which children have many developmental tasks to accomplish and difficulty in achieving them [14]. The age between 9 and 11 years corresponds to the last 2 years of primary school across the world according to the UNICEF (United Nations Children’s Fund) definition [15]. Children at this age are cognitively, emotionally, behaviorally, and socially developing: they build their self-esteem, their mood changes, and they acquire skills in communicating personal experiences and feelings [16]. They have a level of cognitive maturity that enables mental health representations and stigma to develop [17], and they are particularly prone to receive accurate mental health information [18]. At this age, children are a perfect target population for early interventions to modify the trajectory of many mental health issues, prevent progression to more chronic conditions, and promote resilience. Learning proactive coping skills can guide them in the transition into adolescence and middle school, laying the foundation for lifelong positive mental health. Although the incidence of mental illness is high in this age span [19], only a few programs on mental health have been implemented in primary schools [20]. Nonetheless, on an international level, several reports from the World Health Organization (WHO), the Centers for Disease Control and Prevention, and the National Institutes of Health, among others, have advocated for the development and implementation of multisectoral, evidence-informed, and human rights–based interventions for the promotion of mental health and prevention of mental health problems worldwide [21].

### Mental Health Literacy: Definition, Scales, and Interventions

#### Overview

In the field of mental health promotion and prevention, mental health literacy (MHL) is a growing multidisciplinary (psychology, communication sciences, education sciences) theory [22]. It was first conceptualized by Jorm [23] as “knowledge relating to the recognition of a mental health disorder, help-seeking, accessing mental health information, causes and risk factors, professional help and treatments available, self-help strategies, and general attitudes about mental illness.” Kutcher et al [24] have later added to this definition the notion of promoting well-being and developing coping skills. According to MHL, knowledge, information, and understanding are the simplest, quickest, and best ways to overcome mental health problems and enhance positive mental health. Mental health literate people can detect personal and others’ signs of psychological disorders, thus prompting timely help-seeking and access to care. MHL contributes to destigmatizing mental health problems and sensitizing them without any preconceived opinions. Thus, the higher the MHL level, the lower the prevalence of mental health problems [25]. In this sense, MHL is considered a modifiable determinant of mental health [23] because its improvement can contribute to the individual’s empowerment and intervene in mental health behavior change [26].

MHL is defined as “multiconstruct” and “evolving,” meaning that it continuously adjusts depending on the population under study and the context [22]. In this line, in 2020, an Australian team developed an MHL conceptual framework adapted to children below 12 years of age: the Child-Focused MHL model [27]. According to this model, children’s MHL includes 6 dimensions: (1) understanding of mental health and recognition of its fluctuations; (2) help-seeking actions; (3) supports available; (4) influences on mental health; (5) coping and resilience; and (6) stigma.

According to this model, children with good MHL can identify early the signs of distress [28], avoiding late recognition of a mental health problem. MHL encourages help-seeking or the ability to help others promptly. Children can identify the roles of teachers, school nurses and psychologists, pediatricians, and psychiatrists in helping them in case of need. On the one hand, the child learns about risk factors such as difficulties at school, with friends, with the family, or with bullying [29]. On the other, they recognize protective factors such as individual, family, and social resources (optimism and parental support) [30]. Children learn to remain calm, to face their difficulties, and to sympathize with others. Finally, MHL also reduces stigma: mental health problems are common and a child does not have to be ashamed or shame another child.

In its report “The State of the World’s Children 2021 – On My Mind” [31], UNICEF invites all of us to raise MHL to challenge stigma, break the silence about mental health, and ensure the voices of children in distress are heard. According to the Organization for Economic Co-operation and Development (OECD), “good prevention and promotion policies should ensure MHL across populations” [32]. There is thus an urgent need to promote MHL in children before the age of 12 years.

#### MHL Scales

Validated scales are essential for providing precise and reliable data concerning the evaluation of interventions. They have 3 objectives: (1) providing baseline information to monitor health behaviors; (2) providing information for planning and developing an intervention; and (3) raising awareness and improving the agenda for health promotion [33].

Only a few scales are measuring MHL. The very first MHL instrument comprised brief vignettes about people with depression, schizophrenia, attention-deficit/hyperactivity disorder, and anxiety disorder [34]. Individuals were asked to identify the disorder and answer questions about symptoms and treatment. Other measurement tools were quizzes with true/false answers [35]. The Mental Health Literacy Scale (MHLS) [36] is currently the most known tool to measure MHL in the general adult population and includes several close-ended questions and Likert scales.

Concerning young people, there exist only a few scales for adolescents/high school students [37], ranging from telephone interviews [38] to open-ended responses or vignettes [39]. They mostly cover only 1 of the 6 dimensions of MHL [40,41]. There is also no scale for children younger than 12 years, apart from a scale on stigmatization [42] and another scale addressed only to children having a parent with mental illness [43]. Thus, a scale covering all 6 MHL dimensions addressed to primary school children (ages 9-11 years) does not exist so far. Only an attempt has been made by Bale et al [27], but the work has not been finished.

#### MHL Interventions for Young People

To date, most MHL programs have targeted adolescents (12-18 years old), while little attention has been paid to improving MHL among primary school children younger than 12 years. A systematic review synthesized data from 27 articles on MHL interventions in teenagers and young adults [44]. Formats were classroom lectures or presentations, video-watching group discussions, posters, role-playing, drama, internet searching, serious games [45], educational lectures [46], art courses [47], mental health first aid training [48], etc. Concerning the evaluation of their effectiveness, the quality of the evidence was low due to the lack of validated measures and the absence of a robust study design and methodologies (eg, randomized controlled trial [RCT] or mixed methods). Furthermore, there was no collection of data on their acceptability and children’s satisfaction. Identified interventions were only 1 of the dimensions of MHL among either knowledge acquisition or stigmatizing attitudes toward mental illness or help-seeking behaviors. Another systematic review [49] collected information on 20 middle school–based MHL interventions including storytelling and discussions/lectures in class. Once again, evidence of their effectiveness was moderate. Globally, there does not exist any school-based MHL intervention addressed to children aged 9-11 years whose effectiveness has been demonstrated under rigorous scientific conditions.

### Building an Effective Intervention

Several studies [50-52] have shown that school-based interventions have positive effects on children’s well-being. These interventions are inclusive because they are addressed to all pupils without distinction (socioeconomic background or presence of a mental health problem) considering that the large majority of children spend a large amount of their time at school [45,49]. Thus, interventions conducted in this setting can palliate the social gradient enabling children to appraise the concept of mental health and seek help without disparities through an educational approach [53]. In general, the whole-school approach [54] has proved to be effective, as reported by the WHO [55,56]: teachers, school psychologists and nurses, school principals, parents, and children themselves are all involved in promoting organizational MHL [57]. The role of teachers is pivotal. They are the key actors in any school-based intervention. Their implication is essential to the implementation of these interventions.

The effectiveness of interventions is enhanced also by cocreation following the principles of participatory research. The WHO states that collaboration must be initiated from the beginning of the development of interventions as this can contribute to a wider acceptability from the target population and feasibility of the intervention under real-world conditions [58]. To ensure that the intervention is appropriate for the target audience, end users are included in the intervention design and development process. Any other actor in the life of the child is also called upon: for example, teachers, parents, or school psychologists [59].

Theory-based interventions target change in modifiable mechanisms to maximize their effect [60]. Theory-driven approaches can increase our understanding of how a health-related action contributes to an outcome and provide guidelines for producing a successful intervention [61]. The Child-Focused MHL model by Bale et al [27] will be used as the theoretical construct of this project. It explains how knowledge and information build the representations and behaviors of children as far as mental health is concerned. It refers to the mechanisms of actions and beliefs of children for their well-being and those of their entourage. MHL is based on both individual and population-based determinants (eg, socioeconomic status, place of residence, history of a mental health problem) within a specific context explaining the accomplishment of an MHL intervention.

Finally, using several tools (images, videos, or audio) and teaching techniques (transmissive, interactive, experimental, or playful) helps reinforce the transmission of a message, following a communication mix approach [62].

### Objectives, Hypotheses, and Scope

The principal objective of the CHILD-MHL project is 2-fold. It aims to (1) implement an intervention and evaluate its effectiveness concerning children’s MHL and (2) develop and validate a scale measuring children’s MHL.

The CHILD-MHL intervention and the CHILD-MHL scale are the core outputs of the project. They are built in parallel, and they are strongly associated with one another. The effectiveness of the intervention cannot be tested without a scale whose responsiveness can be proved only by an intervention.

The specific objectives are to (1) enhance the effectiveness of the CHILD-MHL intervention using different features—cocreation and design thinking, whole-school approach, theory-based approach, and multitools; (2) outline the CHILD-MHL scale—defining and testing items, administering the scales to a large sample of children, and conducting psychometric analyses for the final validation; and (3) evaluate the effectiveness of the CHILD-MHL intervention using different techniques and methods—performing a viability evaluation, performing a cluster RCT (cRCT), and using mixed methods.

The first hypothesis is that a cocreated, whole-school, theory-based, and multitool intervention makes the intervention itself effective. The second hypothesis is that, to be robust, a scale should be developed through different steps, from an inventory of items (the largest number as possible), their test among the target audience, and the administration in a large sample. The third hypothesis is that strong evidence of the effectiveness of an intervention is provided by the combination of several types of study and the collection of as much data as possible (quantitative and qualitative).

## Methods

### Setting and Participants

This study will be conducted in the department of 1 of the French academies. These are state services that relay, at the local level, the decisions taken by the central administration, the Ministry of National Education and Youth. Each academy is placed under the authority of a rector, appointed by the President of the Republic. For this study, the academy will be selected voluntarily. Each department includes public and private primary schools, in urban, peri-urban, and rural areas. Within each area exist priority education zones (REP) and high-priority education zones (REP+). These schools are considered in need of reinforced pedagogical policies and activities as they face more socioeconomic challenges.

Academies will give access to schools voluntarily (approval by school principals), but we will monitor the inclusion of schools from different geographical and socioeconomic areas and private versus public. We also aim to include localized units for inclusive education. The quota sampling method will be used to ensure the representability of all aforementioned types of schools. All children from recruited classes will participate in the study without any exclusion criteria. Only children whose legal guardians have not given their consent will be excluded. We are targeting children aged 9-11 years.

### Creation: Developing the Intervention and Setting Up the Scale

#### Cocreation of the Intervention

The intervention will be cocreated by different stakeholders: (1) the consortium of the project covering different disciplines (developmental psychology, clinical psychology, educational sciences, child psychiatry, health communication, statistics, public health, and human and social sciences); children; parents; teachers; and 1 designer/artist. Previous reviews on the mechanisms facilitating the effectiveness of school-based interventions will be considered for the development of the intervention [63].

Ahead of the cocreation phase, a survey will be conducted to assess the relevance of an MHL intervention for teachers. An online form will be sent out to teachers from the selected academy. Teachers will then be asked to state whether this intervention is a priority for them and if they can include it in their work plan, among other questions. Some questions will also explore the characteristics that an MHL intervention should have to be easily implemented with positive outcomes.

**Figure 1:**
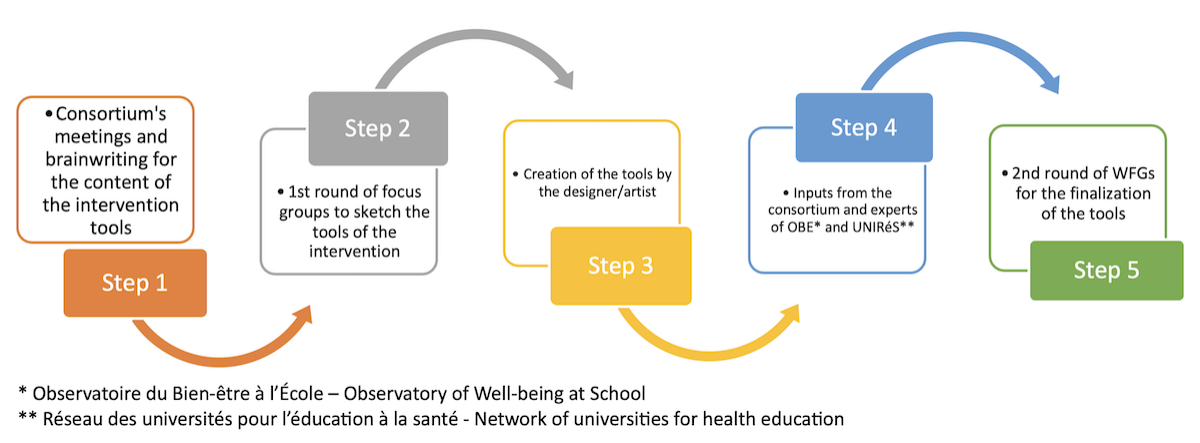
The 5 steps of the co-creation of the CHILD-MHL intervention. MHL: mental health literacy; WFG: working focus group.

The cocreation process will be developed in 5 steps as shown in Figure 1 and Textbox 1.

All working focus groups will last no more than 2 hours to help children focus and pay attention to the tasks.

The entire cocreation process uses the design thinking methodology, involving multiple iterations with all stakeholders until the final production of the tools.

**Steps involved in the cocreation process.**

**Step 1**

The consortium will define the contents of the intervention following the Child-Focused Mental Health Literacy model and covering its 6 dimensions. The consortium will meet online every 2 weeks for 2 months. Using the brainwriting technique (brainstorming through writing and commenting on a shared document), the consortium will produce a report with the key concepts to be transmitted to the target audience.

**Step 2**

During the initial 3 months, a first round of working focus groups (WFGs) will be conducted in 4 classes (priority education zones [REP] or high-priority education zones [REP+], NO-REP, urban, and rural) with a purposive sample of 2 children, 2 parents, 2 teachers, the principal investigator, and 1 designer/artist for each WFG. We plan to have a first round of 4 WFGs, with each corresponding to an end user of the intervention: children, teachers, parents, and school environment (represented by teachers or a school principal). The objective of this step is to transform the contents produced by the consortium into tools adapted to the target audiences. Participants for the WFGs will be recruited voluntarily, and they will draft the first version of the tools, sketching prototypes.

**Step 3**

The designer/artist that participated in the WFGs will collaborate with a colleague to produce mock-ups of the intervention tools based on fieldwork.

**Step 4**

The mock-ups of the tools will be shared with the consortium and other health promotion/education experts and researchers for their feedback.

**Step 5**

After integrating the inputs from the consortium, the tools will be finalized in the second round of the WFGs (3 months), which will take place over 3 months as outlined in step 2.

#### Preparation of the CHILD-MHL Scale

A total of 95 items have already been identified by the founders of the Child-Focused MHL model [27] through a rigorous Delphi study with more than 30 experts. The principal investigator and the research assistant will add items from other MHL scales for adolescents and adults [37,42,64] (inventory of MHL scales) to have a comprehensive list of items necessary to use a reflective model in the definition of the MHL construct. All items will be translated into French and categorized according to the 6 dimensions of the Child-Focused MHL model. We expect a maximum of 150 items.

The consortium will convene online, exchange emails, and use shared documents to streamline the number of items to 60 (ie, 10 per dimension), which corresponds to the first draft of the scale. This number is planned with the objective of limiting the final scale to a maximum of 24-30 items for ease of administration, especially for children who may encounter difficulties due to factors such as limited attention span or the complexity of the tasks. Literature suggests that a preliminary scale must contain 2-3 times the number of items of the final scale and that there must be at least three or four items per dimension [65].

The items will be discussed with a group of 5 parents and 5 teachers to adapt them to children (language and contents). A group of 5 children, who will be recruited from a recreation center (*centre de loisirs*) in a randomly selected area, will then codraw with 1 designer/artist, as a craft activity, the items to obtain a more understandable digital visual scale during a 2-hour workshop. Finally, this scale will be administered to 25 or 30 children from a class randomly selected by a French academy. Each child will be interviewed to assess whether the items are understandable and clear.

In parallel, the same scale will be sent to around 30 experts (eg, psychologists, teachers, health promotion professionals, researchers, and statisticians) who will have to rate each item and complete a checklist measuring the practicality, acceptability, clarity, and appropriateness of the scale.

Qualitative data from children and quantitative data from the checklist will be discussed by the consortium to adapt the items and finalize the scale items. This phase corresponds to the content validity of a scale. This refers to “the degree to which the content of a measurement instrument is an adequate reflection of the construct to be measured” [66], which, in this case, refers to the Child-Focused MHL model.

**Figure 2:**
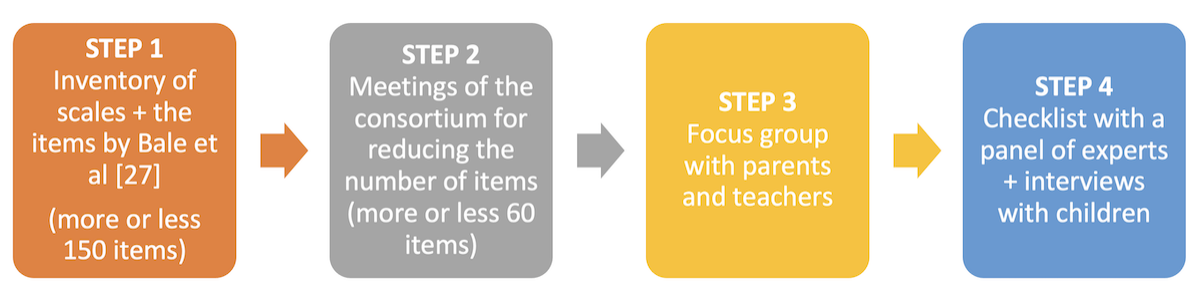
The steps of the content validity of the CHILD-MHL scale. MHL: mental health literacy.

Figure 2 illustrates the 4 steps for defining the items of the scale.

### Refinement: Viability Evaluation of the Intervention and Scale Validation

#### The Viability Evaluation of the Intervention

Conducting a viability evaluation refers to collecting stakeholders’ views and experiences regarding whether the intervention is acceptable, practical, affordable, suitable, integrable, helpful, and sustainable in real-life conditions. For this purpose, a pilot test will be conducted in 4 randomly selected classes, representing various settings (1 REP, 1 rural, 1 urban, and 1 private), within the participating department, involving about 130 children. This number is defined by convenience in an attempt to consider social and territorial disparities.

The principal investigator and the research assistant will monitor how the intervention is carried out and identify the barriers and levers for its implementation. About 15 people among teachers, school psychologists, nurses, school directors, parents, and pupils (purposive sample) will be interviewed for feedback. In particular, the directors of the school will be interviewed to comment on the intervention, its advantages and disadvantages, and whether they think that it can be routinely conducted in their schools. Satisfaction questionnaires will be administered to all participants in the viability study. Data from observations, semistructured interviews, and questionnaires will be analyzed through the mixed methods design.

The intervention (contents, form, and language elements) will also be adapted considering the socioeconomic inequalities. For instance, some tools could be canceled or repeated. Teachers from REP areas will receive extra incentives (salary or learning credits) subject to authorization by the academy.

The viability evaluation conditions will mirror the real implementation of the intervention in the cRCT, with the objective of identifying and addressing potential difficulties and inaccuracies on a tool-by-tool basis.

At this stage, we will define and test the process of recruitment: how to inform schools and teachers (eg, through the academy with flyers, mailing, and videos) and how to obtain the consent of parents (eg, an explanation of the intervention through a detailed booklet, access to all tools for checking their contents). Parents will be informed through school mobile apps, contact book (carnet de liaison), and school meetings. All information will help assess the intervention’s replicability. The viability evaluation will study whether and how teachers can conduct the program autonomously.

This pilot phase is also useful for the implementation of the cRCT because we will record all difficulties encountered while conducting the intervention (eg, access to schools and teachers’ availability).

#### Analysis of Current Policies for Promoting Mental Health in Schools

To verify whether the effectiveness of the intervention is driven by a favorable political environment, we will identify and analyze policies and road maps promoting school-based mental health programs at the micro (department) and macro (France) levels. This mapping will involve approximately 5 semistructured interviews with rectors from various regions and departments, and ideally, some members of the Ministry of National Education and Youth’s cabinet. All collected materials will be synthesized in a narrative review and used to write a policy brief aimed at explaining the need for including MHL interventions in schools in addition to existing mental health–related programs. If the CHILD-MHL intervention effectively increases children’s MHL, we will investigate if this success is emphasized by the local political environment. This might reinforce the need for decision makers to include MHL in all educational policies.

#### Validation of the CHILD-MHL Scale

We will use a specific procedure called COSMIN (Consensus-Based Standards for the Selection of Health Measurement Instruments) [67] for scale validation. The questionnaire including the scale and other variables of interest will be initially administered to children from 10 classes from different schools (ie, around 250 children). Half of these classes are those of the viability evaluation. The sample size of 250 respondents is acceptable, considering feasibility and following the rule of thumb of 10 respondents per item [68]. Besides, feasibility must be considered for data collection. Data will be recorded on a tablet for each child consecutively, with supervision provided by the principal investigator and the research assistant. The research assistant will be the only person in possession of a document containing the child’s name and the corresponding code, allowing for matching the questionnaire administrations. The data from the initial administration will be analyzed to assess the scale’s dimensionality and whether the 60 items adequately cover the 6 dimensions of the Child-Focused MHL model. Exploratory and confirmatory factor analyses will be performed. At this stage, it will be possible for the consortium to add or remove items to cover the 6 dimensions.

After 3 months, the questionnaire with the scale, which might have been modified, will be readministered for the second time to all 250 children from the 10 classes during the week preceding the start of the viability evaluation intervention. This second data collection is essential if the scale has been modified and is highly recommended to refine the scale dimensions. Using this large data set, analyses will be performed to reduce the number of items by employing the Classical Test Theory and the Item Response Theory [69]. Exploratory and confirmatory factor analyses will be performed again to assess the dimensionality and structural validity of the scale. For the convergent and discriminant validity of the scale, the questionnaire will include other variables, including, sex, age, or emotional status measured by the KIDSCREEN-10 [70]. We will check for variations in the scores of scale dimensions among different groups.

Children from the 5 classes of the viability evaluation intervention will complete the questionnaire for the third time after a 3-month interval (ie, the maximum time of the intervention). Data collected from these classes will be used to assess the sensitivity to change or responsiveness of the scale. If the score changes after the intervention, it indicates that the scale can measure the effect of the intervention on children’s MHL. In this case, the items are expected to be “actionable,” meaning that interventions can influence them and lead to changes in the results.

**Figure 3:**
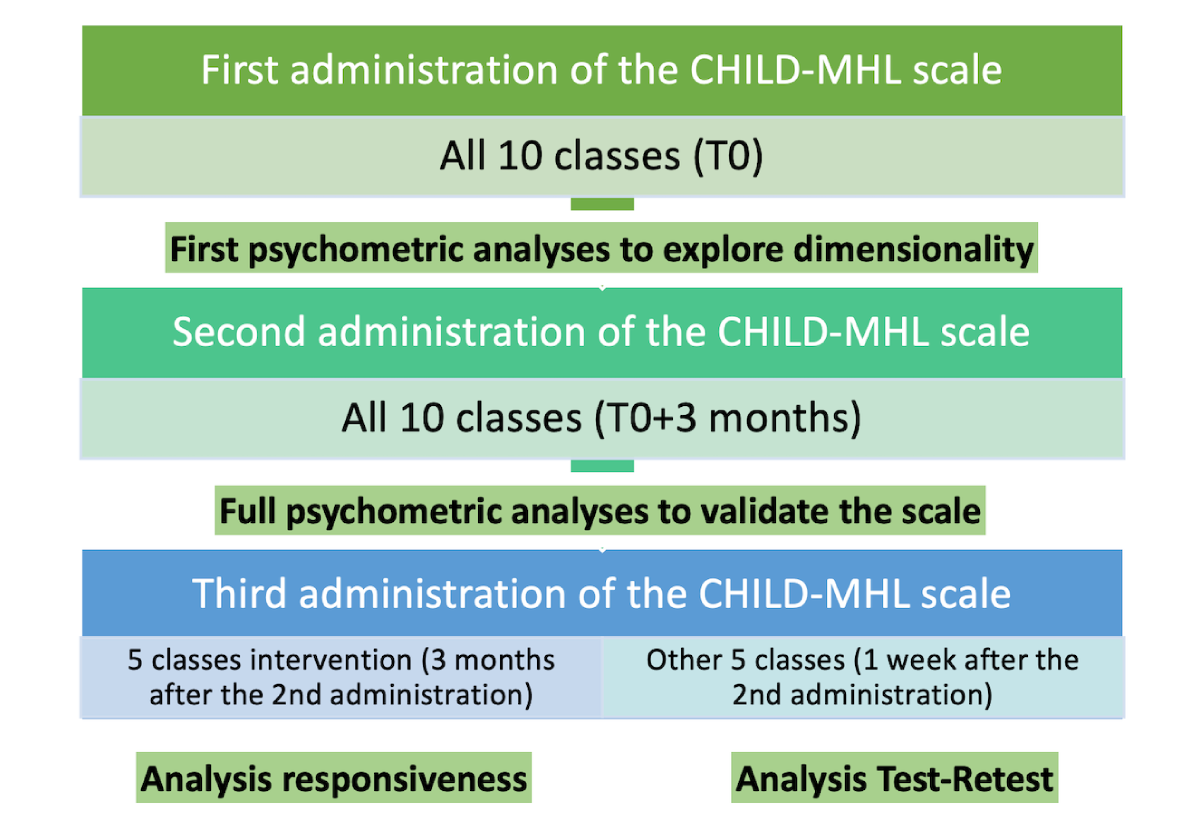
The flow of data collection and analysis for the CHILD-MHL scale. MHL: mental health literacy.

Finally, children not receiving the intervention will answer the questionnaire for the third time after a month. Data will be used to assess the test-retest reliability, determining whether answers remain consistent between administrations and if the items are stable over time. Figure 3 illustrates the phases of the data collection and data analyses for scale validation.

### Assessment: cRCT and Semistructured Interviews

#### Implementing the cRCT

We will conduct the cRCT in a minimum of 16 new classes, representing all typologies under the study, from different schools: 4 REP, 4 rural, 4 urban, and 4 private ones (quota sampling). Feasibility will be a major criterion for participant inclusion. Therefore, a minimum of 400 children will be involved in this small-scale cRCT, which is a substantial sample size, as the literature suggests a minimum of 300 participants is sufficient for a cRCT in children [71]. Furthermore, a limited number of participants will allow us to explore in depth the mechanisms influencing the effectiveness of the intervention by analyzing results for each class.

Classes will be randomly assigned to either the intervention group or the control group (on a waiting list without engaging in any activities). Randomization will involve creating matched pairs based on class types, such as pairing 1 urban school class with the intervention and 1 urban school class without the intervention. The CHILD-MHL scale will be administered 1 week before and 2 weeks after the intervention in all classes. We will collect covariates related to children and their environment, including sex, age, school characteristics (geographical and socioeconomic area, population density), parental background (at least one parent not being French, divorced/separated parents), family size (number of siblings), and self-perceived health and emotional status measured by the KIDSCREEN-10 [70]. If possible, with parental consent, teachers will be asked to report whether there is at least one child with a diagnosed mental health problem in the class and provide information on the parents’ socioeconomic situation. Macro-level data will be also included in the analyses (eg, precariousness indexes). The data from the second administration may offer insights into the intervention’s potential impact on children’s well-being and help-seeking actions. Furthermore, participants who received the intervention will respond to closed-ended questions regarding their intention to help someone with a problem, seek help, and their ability to apply strategies for feeling better and recognizing signs of personal distress. The statistical analysis plan will encompass mixed linear models, multilevel models, descriptive statistics, and random effects, with an intention-to-treat primary analysis.

#### Conducting Semistructured Interviews and Analyzing Data Through Mixed Methods

This phase marks the conclusion of the project, during which we will gather and collate all data to demonstrate the effectiveness of the intervention in improving children’s MHL. We will conduct semistructured interviews with a deliberate selection of 30 pupils and 10 parents from various schools participating in the cRCT. All teachers of involved classes will be also interviewed. These stakeholders will comment on the effects of the intervention on children’s attitude toward mental health: self-perceived benefits of the intervention, what children have learned, and how they will reuse their new knowledge and competencies (from help-seeking to detection of a mental health problem). Interview texts will be coded and treated per theme. Quantitative and qualitative data, collected separately, will be combined and analyzed using a convergent design [72]. When combining the data, we will also consider contextual factors, location, the study population, and other relevant elements that might influence the results. The mixed methods analysis will provide a comprehensive understanding of the intervention.

### Ethics and Regulatory Affairs

Given that children constitute a vulnerable population with limited autonomy, we will implement stringent measures to safeguard their well-being. The CHILD-MHL scale itself should not raise any privacy concerns as it does not collect private or sensitive information. However, variables such as emotional status or self-perceived mental health may introduce potential delays in the procedures. Their inclusion in the questionnaire will be thoroughly justified, but if collecting these data proves challenging, we will rely exclusively on macro-level data (eg, school geographical area, class size) for analysis.

Quantitative data will be collected through a secure tablet-based self-administration software, while qualitative data will be gathered via recorded interviews and subsequently transcribed. We will conduct a Privacy Impact Assessment (PIA) and risk analysis. Additionally, a data management plan will be established to outline data handling procedures throughout and after the project, covering collection, storage, analysis, preservation, and sharing. All collected data will be anonymized.

### Ethics Approval

Ethical approval for the study is going to be obtained from the People Protection Committee (Comité de Protection des Personnes), which is the French Ethical Review Authority. We will strictly adhere to the requirements of the Helsinki Declaration for research involving humans. As a systematic examination of ethical considerations is also needed in the survey study, additional ethical approval will be sought from external researchers. We will make sure that project staff are trained and supervised on ethical issues, with specific attention paid to collecting data from children. In case participants (children, parents, or teachers) have questions, the contact information of the responsible researchers will be provided. In the information letter, study participants will be informed about confidentiality and their right to withdraw from the study at any time. The letter will be adapted for young people. All data will be collected and stored in compliance with the requirements of the Ethical Review Authority and the GDPR (General Regulations for the Protection of Personal Data), as well as the French National Commission on Informatics and Liberty (Commission nationale de l'informatique et des libertés).

## Results

After obtaining the ethical approval, data collection and analyses will be conducted from March 2024. The first paper is expected to be submitted at the beginning of 2025. Finally, the results from all parts of the study will be synthesized in 2026.

We started working on the project in 2022 to predefine some contents and tools. The first tool of the intervention is the pedagogical kit Le Jardin du Dedans, supported by the public organization Psycom Santé Mentale Info and endorsed by UNICEF France. The kit is composed of 12 storyboards introducing MHL to children through an animated story. Each storyboard corresponds to a drawing with specific text. Two manuals are given to educators (eg, teachers, parents) to animate the story and discuss its content with children through tips and key messages. The animation includes 3 sessions conducted in 2 weeks covering the 12 storyboards. Le Jardin du Dedans is based on a metaphor stating that mental health is like an inner garden that we need to take care of across all seasons, from joyful summer to dark winter.

The second tool is a handbook that is addressed to teachers to sensitize them to children’s mental health problems. In 35 pages, teachers are taught the meaning of mental health, the importance of well-being in school, child development and related influences, and main emotional and behavioral disorders (from anxiety disorder to autism spectrum disorder). After reading it, teachers are expected to feel empowered and more confident in dealing with children with mental health problems. The handbook is produced by the Pan American Health Organization and the WHO.

In a 5-page leaflet produced by the nongovernmental organization The Ink Link, children become acquainted with the notion of MHL by discovering its 6 dimensions. Each dimension is illustrated by some examples (eg, the description of mental health professionals and risk and protective factors).

We have also compiled the initial list of items (n=56) for the scale. These 56 items were listed by 2 public health researchers, and revised by 11 experts in the field of child health, including child psychiatrists and developmental psychologists. These items will then undergo further review by a panel of at least 30 experts, including psychologists, educators, and public health researchers. Following this selection process, 1 artist will collaborate with children to illustrate the items. Subsequently, the scale will be administered to several hundred children to collect data for psychometric analyses.

Finally, we compiled a report listing the existing education policies for children’s mental health in France.

## Discussion

### Anticipated Findings

We have started cocreating the intervention to increase children’s MHL. The current partial intervention is composed of 3 tools: a pedagogical kit, a handbook, and a leaflet. In the cocreation process, experts provided the contents of each tool, and artists shaped/transformed them into visually appealing illustrations. These tools were tested with small groups of children and teachers to gather feedback; particularly, the pedagogical kit was animated in several classrooms, the handbook was proofread by teachers, and the leaflet underwent refinement in a classroom setting. The involvement of renewed institutions, namely, UNICEF France, the Pan American Health Organization/WHO, and the nongovernmental organization The Ink Link, ensures a solid foundation for this global intervention [73].

The inclusion of children, teachers, and parents from the outset of the cocreation process proved challenging due to their availability and time constraints, which often conflicted with project funders’ requirements and local service expectations, such as school complexes. These challenges align with findings in the literature [74,75], with logistics and timeline management being the most commonly cited difficulties. However, our study is among the few to employ cocreation with children on a sensitive topic such as mental health, allowing them to play an active role in designing and producing a health-related intervention [76]. Moving forward, our goal is to involve all stakeholders in the cocreation process from the project’s inception.

Few multitool interventions, especially those involving the target audience and the child’s environment, including teachers and parents currently exist [55,77]. Accordingly, we have adhered to a whole-school approach to enhance the tool’s effectiveness. Additionally, it is worth noting that the theory of MHL remains unaddressed in existing interventions.

Usually, only 1 type of support is used (eg, videos or courses). The novelty and uniqueness of our intervention lie in the integration of several tools, potentially enhancing its acceptability and effectiveness [78].

Once all the tools are produced, they will be pieced together and evaluated. Data from viability assessment, cRCT, and interviews will be combined through a mixed methods approach to produce the most accurate evidence [79]. Initial testing of the first tools has shown promising results, particularly with positive feedback from children regarding the pedagogical kit. Furthermore, we have initiated contact with schools to collect data, and the project is gaining recognition within the French academies.

Simultaneously, we have begun developing the scale. Following a rigorous approach, we plan to create an initial version of the scale that aligns with the dimensions of the Child-Focused MHL model. According to the guidelines for scale development [80], the first essential step involves defining a substantial number of items that capture the concept being measured. To ensure content validity (ie, the adequacy with which a measure assesses the domain of interest [81]), we have engaged one of the founders of the Child-Focused MHL model in the item inventory process.

In the end, conducting an inventory of policies in the French academies related to educational programs for children’s mental health is crucial to gauge the acceptability of our intervention. We will assess whether the intervention is more effective in schools with existing mental health programs and a general sensitivity to this topic [82]. Thus, we have initiated a viability evaluation to provide initial evidence on the factors, such as policies, influencing the adoption of the intervention in real-life conditions.

### Limitations

The project is complex, with dual objectives requiring extra effort. Significant human resources are required, particularly for the intervention, which involves recruiting a large number of children. These challenges must be carefully addressed during project planning. However, ambitious studies become feasible with the support of multiple funders (3 are involved in this project, contributing a total of over US $300) and collaboration among researchers from diverse disciplines.

Concerning the cocreation process, the number of participants is small, which might hinder generalizability. However, we hypothesize that the characteristics of the stakeholders involved in our study are representative of children, teachers, and parents in the selected department in France. Therefore, adapting the approach for use in other departments and translating it for other countries will be necessary.

In terms of evaluation, the design we will implement is arduous. While producing robust results, it will be difficult to put into practice. It will require strong logistic capacities and numerous staff members for the cRCT. The multiple semistructured interviews will be time-consuming. Similarly, the statistical analysis of both the evaluation and the scale will require the expertise of 1 or more statisticians for several months.

### Conclusions

Currently, only a few interventions are designed to enhance children’s MHL. Therefore, this project will offer unique insights and practical applications in the field. While the intervention has the potential to reach children in French-speaking countries, its effectiveness must first be demonstrated through rigorous experimental and real-world testing. The project’s findings will inform decisions regarding the extent of implementation efforts. Additionally, this project will pioneer the creation of the world’s first children’s MHLS. Once validated in French, it will be translated into other languages, with English and Spanish taking priority, enabling the measurement of children’s MHL at an international level.

## Abbreviations

COSMIN: Consensus-Based Standards for the Selection of Health Measurement Instruments
cRCT: cluster randomized controlled trial
GDPR: General Regulations for the Protection of Personal Data
MHL: mental health literacy
MHLS: Mental Health Literacy Scale
OECD: Organization for Economic Co-operation and Development
PIA: privacy impact assessment
RCT: randomized controlled trial
REP: priority education zones
REP+: high-priority education zones
UNICEF: United Nations Children’s Fund
WFG: working focus group
WHO: World Health Organization

## Data Availability

The pedagogical kit Le Jardin du Dedans by Psycom Santé Mentale Info, with the support of UNICEF France, is freely available online [83]. The handbook by the Pan American Health Organization and the World Health Organization is also freely available online [84]. The scale items and the list of policies are available upon request. The other tools of the intervention are under construction and will be freely available in the months to come.
